# Clostridia in Premature Neonates' Gut: Incidence, Antibiotic Susceptibility, and Perinatal Determinants Influencing Colonization

**DOI:** 10.1371/journal.pone.0030594

**Published:** 2012-01-26

**Authors:** Laurent Ferraris, Marie José Butel, Florence Campeotto, Michel Vodovar, Jean Christophe Rozé, Julio Aires

**Affiliations:** 1 Université Paris Descartes, EA4065, Faculté des Sciences Pharmaceutiques et Biologiques, Paris, France; 2 Néonatalogie, Institut de Puériculture, Paris, France; 3 Université de Nantes, Département de Médecine Néonatale, Centre d'Investigation Clinique INSERM CIC004, CHU de Nantes, Nantes, France; Université d'Auvergne Clermont 1, France

## Abstract

**Background:**

Although premature neonates (PN) gut microbiota has been studied, data about gut clostridial colonization in PN are scarce. Few studies have reported clostridia colonization in PN whereas *Bacteroides* and bifidobacteria have been seldom isolated. Such aberrant gut microbiota has been suggested to be a risk factor for the development of intestinal infections. Besides, PN are often treated by broad spectrum antibiotics, but little is known about how antibiotics can influence clostridial colonization based on their susceptibility patterns. The aim of this study was to report the distribution of *Clostridium* species isolated in feces from PN and to determine their antimicrobial susceptibility patterns. Additionally, clostridial colonization perinatal determinants were analyzed.

**Results:**

Of the 76 PN followed until hospital discharge in three French neonatal intensive care units (NICUs), 79% were colonized by clostridia. *Clostridium* sp. colonization, with a high diversity of species, increased throughout the hospitalization. Antibiotic courses had no effect on the clostridial colonization incidence although strains were found susceptible (except *C. difficile*) to anti-anaerobe molecules tested. However, levels of colonization were decreased by either antenatal or neonatal (during more than 10 days) antibiotic courses (p = 0.006 and p = 0.001, respectively). Besides, incidence of colonization was depending on the NICU (p = 0.048).

**Conclusion:**

This study shows that clostridia are part of the PN gut microbiota. It provides for the first time information on the status of clostridia antimicrobial susceptibility in PN showing that strains were susceptible to most antibiotic molecules. Thus, the high prevalence of this genus is not linked to a high degree of resistance to antimicrobial agents or to the use of antibiotics in NICUs. The main perinatal determinant influencing PN clostridia colonization appears to be the NICU environment.

## Introduction

Clostridia are gram-positive endospore-forming obligate anaerobes, found in the environment, and are common inhabitants of the human and animal intestinal microbiota. This genus is of medical importance as many species are responsible for several diseases in adults and children. Clostridial infections are either from exogenous sources such as food-borne diseases (*Clostridium perfringens* or *Clostridium botulinum*), wound infections (*C. perfringens*), hospital-acquired infections (*Clostridium difficile* antibiotic associated diarrhea or pseudomembranous colitis), or from the host's endogenous microbiota [1]. These latter clostridial infections, often in association with various bacterial species, tend to be more severe than comparable infections without clostridia [1]. In neonates, asymptomatic carriage of toxigenic or non toxigenic clostridia in the digestive tract has been reported, but it may also be associated with infections [2], [3]. The incidence of anaerobes in neonatal bacteraemia varies between 1.8 and 12.5%, among which about one third are due to clostridia, mostly *C. perfringens* [3].

Clostridia are supposed to be involved in necrotizing enterocolitis (NEC) in premature neonates (PN), a devastating gastrointestinal disease with high morbidity and mortality [4]–[9]. However, recent data did not confirm clostridia and NEC association [10]. Nevertheless, *C. perfringens*, *Clostridium butyricum*, or *Clostridium paraputrificum* have been isolated from the blood, feces, and peritoneal fluids of PN suffering from NEC [5], [11]–[13]. Early gut colonization by *C. perfringens* has been associated with NEC [14], [15] and *Clostridium neonatale* with a NEC outbreak [7]. Moreover, *C. butyricum*, *C. perfringens* and *C. paraputrificum* were shown to be responsible for cecal NEC-like lesions through bacterial fermentation end-products in quail animal model of NEC [16]. More recently, Smith et al. [17] reported a correlation between the presence of *C. butyricum* and *C. paraputrificum* and pneumatosis intestinalis in tissue specimens from PN with NEC. All together, these reports suggest that clostridia could be either primary pathogens or secondary invaders in NEC. However, despite similarities between NEC and clostridial infection, isolation of clostridia is rarely performed routinely in PN.

If PN gut microbiota has been studied, data about gut clostridial colonization in PN are scare. Few studies have reported clostridia colonization in PN whereas *Bacteroides* and bifidobacteria have been seldom isolated [18]–[20]. Such aberrant gut microbiota has been suggested to be a risk factor for the development of intestinal infections such NEC [21], [22]. Besides, PN are often treated by broad spectrum antibiotics, but little is known about how antibiotics can alter clostridial colonization based on their susceptibility patterns. The aim of this study was therefore to report the distribution of *Clostridium* species isolated in feces from PN and to determine their antimicrobial susceptibility patterns. Statistical analysis was performed to investigate clostridial colonization perinatal determinants.

## Materials and Methods

### Study and population

This retrospective study included 76 PN born in three independent French neonatal intensive care units (NICUs) from 2002 to 2007. PN in this study had been included in random controlled trials (with control groups) to evaluate the effects of premature neonatal formula, which have been demonstrated to have no effect on intestinal bacteria colonization [18], except for colonization by the two probiotic strains [19]. In those studies, to be eligible for enrolment, PN must have been born at gestational age (GA)<36 weeks (wk) and without any malformations or metabolic diseases. Written informed parental consent was obtained for each PN before inclusion. The protocol was approved by the local Institutional Review Board [18], [19]. PN were enrolled during their first 3 days of life until hospital discharge.

### Microbiological analysis of fecal samples

Fecal samples, collected weekly during hospitalization from diapers, were placed in sterile tubes, frozen and kept at −80°C until analyzed. Fecal microbiota analysis was performed using culture and PCR-temporal temperature gradient electrophoresis, as in previously studies [18], [19]. For quantitative analysis of clostridia, 0.1 to 0.5 mg of frozen fecal samples were crushed in Brain Heart Infusion broth using an Ultra-Turrax T25 (Fisher-Bioblock, Illkirch, France) apparatus. After dilution in peptone water, 10^−2^, 10^−4^ and 10^−6^ dilutions were spread using the WASP® apparatus (AES Chemunex, Bruz, France) on cysteine (160 mg/L) Columbia agar base supplemented with sheep blood 5% [23], and whole milk 5%, colistin (10 mg/L) and neutral red (40 mg/L) for all clostridia [24] and *C. difficile* supplement (bioMérieux, Marcy l'Etoile, France) for *C. difficile*. Media incubation was performed for 48 h at 37°C in an anaerobic chamber (MACS®, AES Chemunex) under anaerobic gas phase (H2∶CO2∶N2, 10∶10∶80, vol/vol/vol). Bacterial counts were expressed as the log_10_ colony forming units (CFU)/g of feces, and the count threshold was 3 log_10_ CFU/g of feces.

### Strain identification

Colonies that were able to grow on the selective medium and suspected to be clostridia on the basis of cellular morphology and Gram staining were identified using Rapid ID 32A strips (bioMérieux). Identification of each isolate was confirmed by partial sequencing of 16S rRNA gene, which was amplified by PCR using primers LPW58 (5′-AGGCCCGGGAACGTATTCAC-3′) and LPW81 (5′-TGGCGAACGGGTGAGTAA-3′) [25]. PCR conditions were as follows: PCR mixture was composed of 1 µM of each primer, 5% of DMSO, each deoxynucleotide triphosphate at a concentration of 250 µM in 1× PCR buffer and 1.25 U of recombinant DNA polymerase (Invitrogen, Illkirch, France) in a final volume of 25 µl. The PCR program was 30 s at 95°C, followed by 40 cycles of 1 min at 94°C, 1 min at 55°C, 2 min at 72°C and a 10 min final extension at 72°C. PCR products were then sequenced (Beckman Coulter Genomics, Takeley, United Kingdom).

### Antibiotics susceptibility testing

Clostridia antibiotic susceptibility testing was performed using the disk-diffusion method on Brucella agar medium supplemented with 5% of laked sheep blood and 1 µg of vitamin K1/mL according to the recommendations of the Comité de l'Antibiogramme de la Société Française de Microbiologie [26]. The antibiotic disks (bioMérieux) used were amoxicillin, amoxicillin-clavulanic acid, piperacillin, piperacillin-tazobactam, ertapenem, imipenem, cefoxitine, cefotaxim, clindamycine, tetracycline, tigecycline, chloramphenicol, moxifloxacin, metronidazole, linezolide, and vancomycin (Bio-Rad, Marnes la Coquette, France). When necessary, MICs for amoxicillin, piperacillin, piperacillin/tazobactam, ertapenem, cefotaxim, metronidazole, tetracycline, and tigecycline were determined using E-test strips as specified by the manufacturer (bioMérieux).

### Statistical analysis

Statistical analysis was performed using SPSS® 15.0 software. Student's t test, or Mann-Whitney when appropriate, were used for comparison of continuous variables and Chi-2 test or Fisher's exact test when appropriate, was used for comparison of categorical variables. Univariate analysis and multivariate regression analysis were used to evaluate the relationship between perinatal (first week of life) and neonatal characteristics (at hospital discharge), i.e. GA, mode of delivery, antenatal, intrapartum or neonatal antibiotic treatments, and NICUs. All tests were two-tailed and p-values less than 0.05 were considered significant.

## Results

### Population characteristics

Table 1 lists the characteristics of the 76 PN included in this study. The PN were born at a median gestational age of 32.8 wk [interquartile, 29–34 wk] and had a median birth weight of 1695 g [interquartile, 1313–2103 g]. They were followed from birth until hospital discharge (median 4 wk, [interquartile 2.3–5.0 wk]). No PN were suffering from NEC.

**Table 1:** Characteristics of the premature neonates included in the study.

Population	*n* = 76 (Range)
Gestational age, weeks	31.0±3.3 (24.0–35.9)
Birth weight, g	1656±543 (550–2750)
Delivery mode	
vaginal delivery	41
caesarean section	35
Maternal antibiotic therapy	
antenatal	29
intrapartum	32
Neonatal antibiotic therapy	41
duration, days	9.6±10.4 (1–41)

Among the 76 PN, 29 PN (38.2%) were born from mothers who received antenatal treatment within 8 days before birth with the following antibiotics used either alone or in combination: amoxicillin (82.7%), gentamicin (27.6%), metronidazole (10.3%), clindamycin (6.9%), amoxicillin-clavulanic acid (6.9%), and not documented (17.2%). Thirty-two PN (42.1%) were born from mothers receiving intrapartum antibiotic therapy using: amoxicillin (87.5%), gentamicin (56.2%), cefixime (3.1%), cefazoline (3.1%), erythromycin (3.1%), and not documented (9.3%). Forty-one PN (54%) underwent neonatal antibiotic therapy for suspected (*n* = 17) or confirmed infection (*n* = 24). Prescriptions were administered for a median of 5 d [interquartile 2–11.5 d] and comprised amoxicillin (80.5%), cefotaxime (85.4%), aminoglycoside (gentamicin or amikacin) (80.5%), vancomycin (29.3%), metronidazole (9.7%), macrolide (josamycin or erythromycin) (9.7%), imipenem (7.3%), and oxacillin (2.4%).

### Clostridial colonization

**Figure 1:**
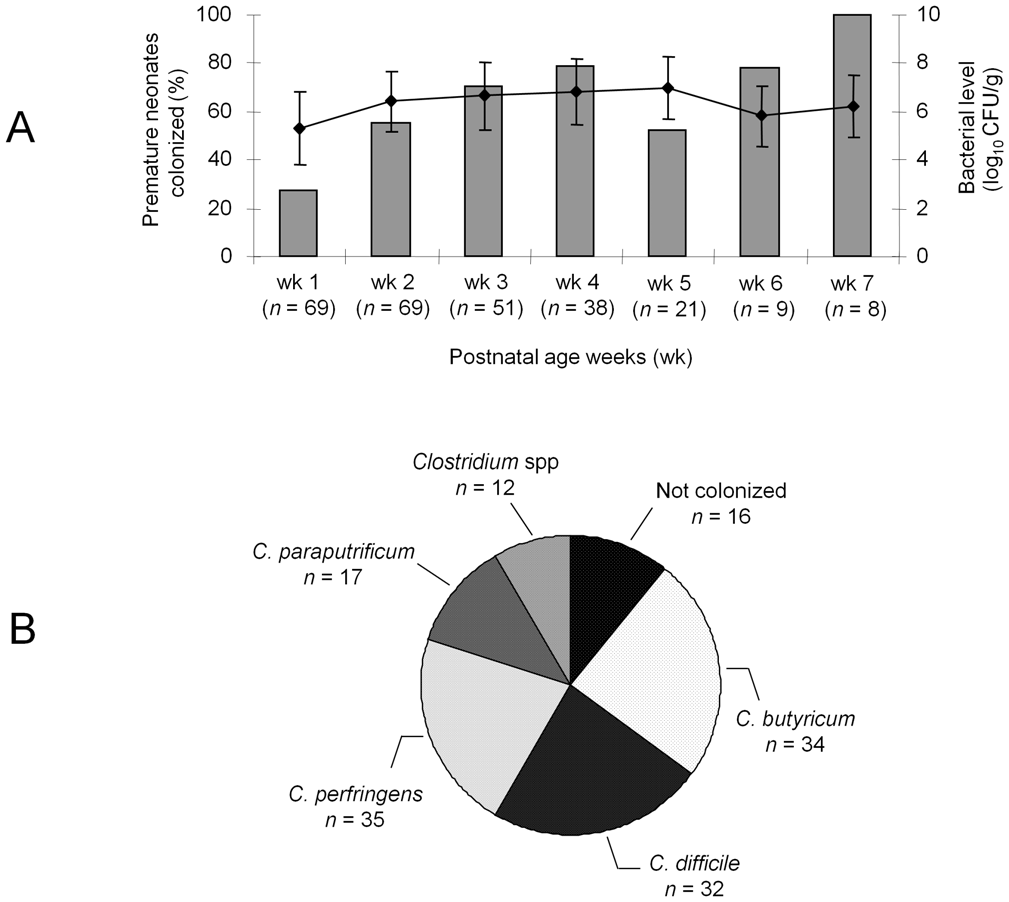
Clostridial colonization of the premature neonates included in the study. **A**. Clostridial colonization of the premature neonates (*n* = 76) during the first 7 weeks of hospitalization. Bar graphs indicate percentage of colonized premature neonates by clostridia. Line graph indicate mean levels of colonization by clostridia (log_10_ colony-forming unit (CFU)/g of feces), with standard deviation represented by vertical bars. **B**: Number of premature neonates colonized by the different *Clostridium* species. *Clostridium* spp included *C. baratii* (*n* = 3), *C. tertium* (*n* = 3), *C. disporicum* (*n* = 2), *Robinsoniella peoriensis* (*n* = 2) *C. glycolicum* (*n* = 1) or *C. orbiscindens* (*n* = 1).

In the current study, of the 271 fecal samples analyzed (average of 3.6 samples per neonate), 237 clostridia strains were isolated and corresponded to 130 non redundant *Clostridium* strains. Non redundant strains were defined as strains isolated from one individual and belonging to one species. At hospital discharge, 60 out of the 76 PN (78.9%) were colonized with clostridia species at levels ranging from 3.3 to 9.2 log_10_ CFU/g feces (Fig. 1A). The proportion of PN colonized by *Clostridium* sp increased throughout the hospitalization period: 27.5% were colonized after the first week of life, 78.9% at wk 4, and 100% of the PN hospitalized after wk 7 (Fig. 1A). PN were colonized with one (*n* = 18), two (*n* = 23), three (*n* = 10), or up to 4 (*n* = 9) different clostridia species. The most frequently recovered species were *C. perfringens* (46.1% of the PN), *C. butyricum* (44.7%), *C. difficile* (42.1%), and *C. paraputrificum* (22.4%). Other recovered species were as follows: *C. baratii* (3.9% of the PN), *C. tertium* (3.9%), *C. disporicum* (2.6%), *Robinsoniella peoriensis* (2.6%) *C. glycolicum* (1.3%) or *C. orbiscindens* (*Flavonifractor plautii*) (1.3%) (Fig. 1B).

### Clostridial antimicrobial susceptibility

Antimicrobial susceptibility levels of all the isolates (*n* = 237) was performed. Table 2 presents the data for the 139 non duplicate strains. Duplicate strains were defined as strains isolated from the same PN and belonging to the same bacterial species and for which no major discrepancies in antibiotic susceptibility (susceptible versus resistant, or the reverse) were found throughout the study period. All the strains tested were susceptible to amoxicillin-clavulanic acid, piperacillin-tazobactam, chloramphenicol, metronidazole, linezolid, and vancomycin. Overall, strains were resistant to clindamycin (51.8% of the strains), cefotaxim (38.1%), tetracycline (17.3%), and moxifloxacin (1.4%) (Table 2). Intermediate phenotypes to moxifloxacin, cefotaxim and imipenem represented respectively 49.6, 18.0, and 1.4% of the strains (Table 2). Resistance to cefotaxim was observed for all *C. difficile* strains and 42.5% of the *C. butyricum* strains. In addition to cefotaxim, some of the *C. butyricum* strains were resistant to amoxicillin (7.5%) and piperacillin (12.5%) (Table 2). Based on the nitrocefin assay, these latter resistant strains (*n* = 5) showed a β-lactamase. Resistance to clindamycin was observed for *C. difficile* (97.0% of the isolates), *C. paraputrificum* (94.1%), *C. perfringens* (40.5%), and *C. butyricum* (2.5%) (Table 2). Tetracycline resistance was distributed among strains of *C. perfringens* (32.4% of the strains), *C. butyricum* (25.0%), and *C. difficile* (3.0%), and in addition 8.0% of the *C. perfringens* strains were resistant to tigecyclin (Table 2). Concerning moxifloxacin, 94.5% of the *C. perfringens* isolates showed an intermediate phenotype. Although, there was no resistance observed to moxifloxacin, 39.4% of *C. difficile* strains were found intermediate (Table 2).

**Table 2:** Antibiotic resistance phenotypes of the 139 non duplicate clostridia strains isolated from the feces of the 76 premature neonates included in the study.

	Percentage of strains^a^		
Isolates (number tested) and antibiotics	S	I	R^b^
*Clostridium perfringens* (*n* = 37)			
clindamycin	59.5		40.5
tetracyclin	19	48.6	32.4
tigecyclin	86.5	5.5	8.0
moxifloxacin	5.4	94.6	
*Clostridium difficile* (*n* = 33)			
imipenem	93.9	6.1	
ertapenem			100
cefoxitin			100
cefotaxim			100
clindamycin	3.0		97.0
tetracyclin	94.0	3.0	3.0
moxifloxacin	60.6	39.4	
*Clostridium butyricum* (*n* = 40)			
amoxicillin	87.5	5.0	7.5
amoxicillin/clavulanic acid	100		
piperacillin	87.5		12.5
cefotaxim		57.5	42.5
clindamycin	97.5		2.5
tetracyclin	75.0		25.0
moxifloxacin	77.5	22.5	
*Clostridium paraputrificum* (*n* = 17)			
clindamycin	5.9		94.1
moxifloxacin	64.7	35.3	
*Clostridium* spp^c^ (*n* = 12)			
cefotaxim	58.3	16.7	25.0
clindamycin	33.3		66.7
tetracyclin	91.7		8.3
moxifloxacin	33.3	50.0	16.7
Total of *Clostridium* strains (*n* = 139)			
amoxicillin	96.4	1.4	2.2
piperacillin	96.4		3.6
imipenem	98.6	1.4	
ertapenem	76.3		23.7
cefoxitin	76.3		23.7
cefotaxim	43.9	18.0	38.1
clindamycin	48.2		51.8
tetracyclin	69.0	13.7	17.3
tigecyclin	96.4	1.4	2.2
moxifloxacin	49.0	49.6	1.4

a Antibiotic susceptibility testing was performed using the disk-diffusion method. The antibiotic disks used were amoxicillin, amoxicillin-clavulanic acid, piperacillin, piperacillin-tazobactam, ertapenem, imipenem, cefoxitine, cefotaxim, clindamycine, tetracycline, tigecycline, chloramphenicol, moxifloxacin, metronidazole, linezolide, and vancomycin.

Data corresponding to 100% susceptibility among strains of one species are not presented.

b S, susceptible ; I, intermediate ; R, resistant.

c *Clostridium* spp: *C. baratii* (*n* = 3); *C. tertium* (*n* = 3); *C. disporicum* (*n* = 2); *Robinsoniella peoriensis* (*n* = 2); *C. glycolicum* (*n* = 1); *C. orbiscindens* (*n* = 1).

### Analysis of clostridial colonization perinatal determinants

Statistical analysis showed no relationships between the incidence of intestinal clostridial colonization at wk 1 or at hospital discharge and either GA, or birth weight, or delivery mode, or antibiotic courses. However, the incidence tended to be higher in PN born at a GA less than 29 wk (p = 0.07). Besides, the incidence of colonization was higher for PN born in one of the three NICUs participating to the study. After multivariate logistic regression analysis, only NICU remained independently associated with the incidence of colonization (p = 0.048) for the NICU mentioned above.

**Figure 2:**
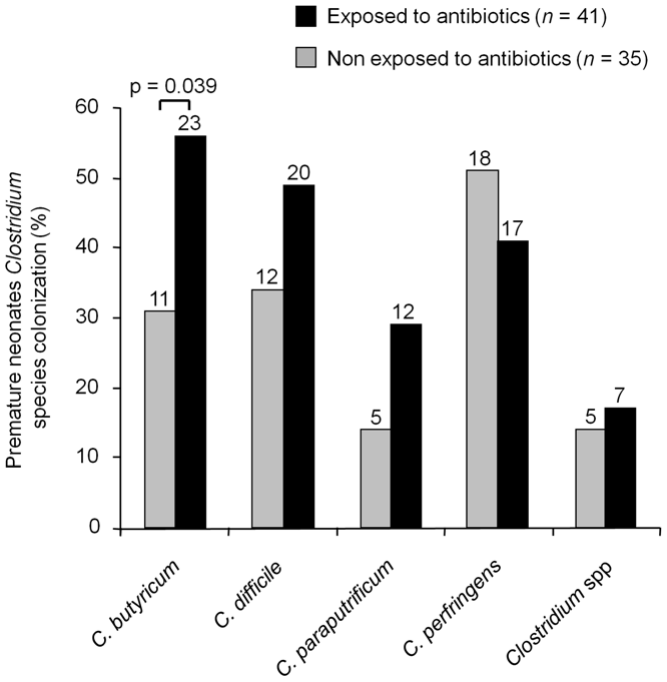
Comparison of clostridia species recovery in antibiotic exposed and non exposed premature neonates included in the study. Numbers correspond to the non redundant strains isolated (non redundant strains were defined as strains isolated from one individual and belonging to one species).

Comparison of clostridia recovery among antibiotic exposed and non exposed PN included in the study are presented in Fig. 2. These data confirm the absence of effect of antibiotics on the overall clostridial colonization. However, as far as species are concerned, we observed a significant higher frequency of *C. butyricum* isolation (p = 0.039) in fecal samples from PN previously exposed to antibiotics.

When concidering clostridia colonization levels, antenatal antibiotic treatment significantly decreased clostridia levels (p = 0.006), while intrapartum antibiotic therapy had no effect. Neonatal antibiotic therapy significantly decreased the levels (p = 0.001) of clostridial colonization only when performed during more than 10 days (*n* = 11 PN).

## Discussion

The present study reports, for the first time, the incidence of clostridia species colonizing the gut of PN and the perinatal determinants influencing clostridial colonization. Statistical analysis showed that the NICU was a major determinant influencing PN clostridia colonization and that antibiotic courses influenced the levels of colonization.

This study reports a mean high incidence in clostridial colonization during hospitalization, since about 80% of the PN were colonized. Additionally, we showed a sharp increase in this incidence throughout the hospitalization period: all the PN have been colonized after wk 7. Studies using either culture [27]–[30] or culture-independent [20], [31], [32] methods previously reported incidences of colonization ranging from 20–60% and 7–40%, respectively. Likewise, increase in clostridia colonization throughout the study period has been previously described by culture (incidences of 30% at wk 4 of life [29]) or culture-independent methods (incidence of 45% at wk 7 of life [20] and 32% at 1month of life [32]). The high incidence of clostridial colonization that we observed compared to these previous studies may suggest possible differences in either the studied population in terms of perinatal characteristics, or nursing conditions, or hospital environment.

A high diversity in species colonizing PN was observed in the present work. The most frequent species isolated were *C. perfringens*, *C. butyricum*, *C. difficile*, and *C. paraputrificum* with fluctuations in their relative levels. The first three species were previously isolated in quite similar incidences by culture methods [27], [29]. By contrast, *C. paraputrificum* was rarely isolated. Comparison of our data with results obtained through culture-independent studies is difficult as little data are available concerning clostridia colonization. Indeed, culture-independent approaches described clostridia identification either at the phylum, order, family or genus levels [10], [33], [34]. Some studies did not find clostridia at all [35]–[37]. Most of the approaches were based on PCR techniques using universal primers or primers designed to amplify the variable 16S rRNA gene regions. However, the design of primers is challenging because clostridia belongs to a complex phylogenetic heterogeneous group [38], [39]. For instance, *C. difficile* belongs to the cluster XI of the non group I of *Clostridium* species while *C. butyricum*, *C. perfringens*, or *C. paraputrificum* belong to the cluster I of the group I of *Clostridium* species [39]. Additionally, for one cluster a high genetic diversity among the different strains was reported: *C. perfringens* and *C. butyricum* representative strains were distributed respectively on 9 and 6 different clusters [38]. Therefore, future studies on clostridia will need a careful analysis of 16S rRNA sequences in order to design specific and discriminant probes in order to search for specific clostridia species.

Quantitative and qualitative analysis of the faecal microbiota on the PN had been previously described and it showed: (i) high incidences and levels of staphylococci colonization; (ii) a more or less delay in colonization by enterobacteria and enterococci; (iii) a sharp delay in colonization by anaerobic genera (except clostridia) (for details see [18], [19]). In this study we showed that clostridia are part of the autochthonous gut microbiota of PN. It raises the question of the risk of clostridia gastro-intestinal induced pathologies. Indeed, such high incidence of clostridia associated with the delay in colonization by other anaerobes [18]–[20] leads to a dysbiosis of the microbiota in PN that might have health consequences [37]. The most frequently isolated species - *C. perfringens*, *C. butyricum*, *C. paraputrificum* - have been implicated in NEC [37]. But despite NEC's clinical similarity to clostridial infection [8] and evidence through animal studies [16], only few studies searched for anaerobic bacteria. More recently, culture-independent techniques did not identify significant differences in the fecal microbiota of PN with and without NEC [35], [40], or reported diminished overall microbial diversity and abundance [33], [41]. One report associated early colonization by *C. perfringens* and NEC [15]. Alternatively, novel non identified pathogens might contribute to the aetiology of NEC [10]. However, limitations of these culture or culture-independent studies were either the small number of cases [10], [15], [33] or the collection of samples had been obtained after onset of the disease and initiation of antibiotics treatment [35], [40] which limits interpretations. Moreover, culture-independent techniques did not allow identification of bacteria in a subdominant status although it can have harmful biological effects. While involvement of clostridia in NEC onset remains questionable [10], it is consistent with the high concentrations of short chain fatty acids, excessive luminal gas production, and pneumatosis that are associated with NEC lesions [16], [17], [42]. However, since we showed that clostridia belong to the commensal microbiota of PN, NEC might be induced by likely “NEC-associated” strains that could display unknown virulence factors associated with the highly immunoreactive intestinal mucosa of PN [9].

Fear of PN infections often leads to early use of empiric broad-spectrum antibiotics at the NICUs, a strategy that increases the risk of colonization with resistant strains [43]. In this study, antibiotic courses decreased clostridial colonization levels but had no effect on the colonization incidence. The lack of a strong effect of antibiotics could not be explained by the resistance pattern of the clostridial strains. Indeed, *C. perfringens* strains were susceptible to almost all molecules tested (except to clindamycin, tetracycline and tigecycline). *C. difficile* resistance levels to cefoxitin, cefotaxim, ertapemen, clindamycin and susceptibility levels to imipenem and tetracycline were as previously reported for strains isolated from hospitals [44]–[46]. No resistance was observed to moxifloxacin, although it has been described at high frequencies [46], but one third of our strains were found to be intermediate. Resistance to amoxicillin and piperacillin was observed for *C. butyricum* strains and was attributed to its specific β-lactamase inhibited by clavulanic acid [47]. Nevertheless, whereas antibiotic courses did not influence overall clostridia colonization, the PN exposed to antibiotics were more often colonized with *C. butyricum* species.

In the current study, statistical analysis was performed to test whether perinatal determinants could be related with clostridial colonization. By contrast with full-term neonates [48]–[50], delivery mode had no effect on clostridial colonization. This confirms the previous reports on the limited influence on initial colonization in PN delivered through caesarean section [20], [51]. In our study, the perinatal determinant influencing clostridia colonization appears to be the NICU. The fact that clostridial colonization incidence increased during the hospitalization suggests a colonization from the environment. Indeed, clostridia, despite being strict anaerobes, are spore-forming bacteria, which can survive in hospital environment, such as previously demonstrated for *C. difficile* [52]. The high influence of the environment is in accordance with the study of Schwiertz et al. [36] who reported an increase in the similarity of the bacterial communities of PN during their hospital stays.

In conclusion, this study shows that, by contrast with other anaerobes, clostridia are part of the PN gut microbiota. The high prevalence of this genus in PN is therefore not linked to a high degree of resistance to antimicrobial agents and to the wide use of antibiotics in NICUs. The perinatal determinant influencing PN clostridia colonization appears to be the NICU environment. This study gives new insights on the factors of colonization of clostridia species and may participate to better understand neonatal gastrointestinal infections involving clostridia.
